# Limitations of the Check Calculation for Tooth Deformation of Plastic Gears According to Gear Design Guideline VDI 2736

**DOI:** 10.3390/polym15183809

**Published:** 2023-09-18

**Authors:** Christoph Herzog, Dietmar Drummer

**Affiliations:** Institute of Polymer Technology (LKT), Friedrich-Alexander Universität Erlangen-Nürnberg, 91054 Erlangen, Germany; dietmar.drummer@fau.de

**Keywords:** tooth deformation, plastic gear design, in situ gear testing

## Abstract

An in situ gear test rig has been developed at the Institute of Polymer Technology (LKT) to characterize the deformation of plastic gears during operation. It analyses timing differences between following index pulses of rotary encoders on the input and output shaft. This measurement principle enables the continuous measurement of the elastic tooth deformation and permanent deformations and wear at operating speed by switching between a high and low torque. Gear tests using a steel-polybutylene terephthalate (PBT) gear set were performed at different rotational speeds and tooth temperatures to analyze the tooth deformation during operation. The results were compared to the calculated deformation according to gear design guideline VDI 2736. Moreover, the results of the gear tests were correlated with the results of a dynamomechanical analysis (DMA). Both, the DMA and the in situ gear tests show that the effect of temperature on deformation is much higher than the effect of frequency or rotational speed. However, the experimentally measured tooth deformation is significantly higher (up to 50%) than the calculated at lower speed. Thus, the check calculation according to VDI 2736 underestimates the actual tooth deformation at lower speeds. Therefore, the guideline should be adjusted in the future.

## 1. Introduction

Thermoplastic gears show many advantages compared to metal gears due to the specific properties of polymers. These are, for instance, noise reduction [1], dry run capability [2], light weight, the possibility of material modification by fillers [2] and economic production by injection molding [3]. As a consequence, plastic gears are widely used in different industries, such as automotive, medical engineering and home appliances [3]. Examples of practical applications are actuating drives in cars, medical devices such as drug dosage systems or drives for home appliances such as printers. On the downside, the properties of the thermoplastic material lead to specific failure modes such as melting, tooth flank wear and tooth deformation [4]. This has to be taken into account when designing plastic gear sets for industrial applications. Due to the lower mechanical strength and stiffness of plastic parts, the plastic gear’s teeth are bent during operation, which leads to meshing impacts and unfavorable stress situations [5].

However, until now, the current design guideline VDI 2736 [4] has not considered all relevant aspects of the material behavior of polymers. Especially the influence of temperature and load frequency on the mechanical and tribological properties is not sufficiently taken into account. Moreover, there is barely any experimental data covering the tooth deformation of plastic gears in the literature due to the lack of appropriate test methods. To enable a cost- and resource-efficient design of plastic gears, the design guideline VDI 2736 has to be improved. Therefore, this work provides an investigation of the influence of rotational speed and temperature on tooth deformation of plastic gears during operation using a novel test rig concept. In addition, the experimental results are compared to the calculated tooth deformation according to gear design guideline VDI 2736.

### 1.1. Tooth Deformation of Plastic Gears

VDI 2736 Part 2 contains design and check calculation formulas for the determination of the load-carrying capacity of plastic gears. Apart from the tooth deformation calculation, there are also suggestions for the calculation of the tooth root load-carrying capacity, the frictional wear load capacity and the tooth temperature. Moreover, VDI 2736 Part 2 provides a check calculation formula for peak loads.

According to VDI 2736 Part 2 [4], the teeth of plastic gears are deformed because of the low elastic modulus of polymers. At the transition from the loaded to the unloaded tooth, the deformation acts as a pitch error. VDI 2736 Part 2 describes the deformation of the tooth tip in the circumferential direction λ as follows [4]:

(1) $$\lambda =\frac{{7.5\cdot F}_{t}}{b\cdot \cos\beta }\cdot \left(\frac{1}{E_{1}}+\frac{1}{E_{2}}\right)$$

The tooth deformation λ depends on the nominal tangential force $F_{t}$, which is the tangential force at the pitch circle diameter, the face width b, the helix angle β, which is 0° for spur gears, and the elastic modulus of the pinion ($E_{1}$) and plastic gear ($E_{2}$). A guide value for the maximum allowed deformation in terms of running noise and lifetime is [4]:

(2) $$\lambda_{zul}=0.07\cdot m_{n}$$

**Figure 1:**
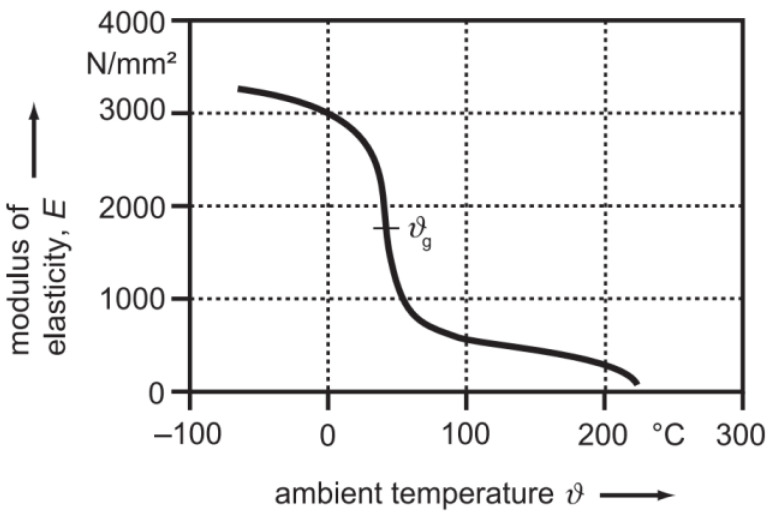
Elastic modulus plotted against temperature measured via dynamomechanical analysis under torsional/flexural stress for small loads according to VDI 2736 Part 1 [6].

VDI 2736 Part 1 [6] provides material data for the temperature-dependent elastic modulus of certain relevant polymers, as seen in Figure 1 for PBT, which is suggested to be used for the calculation of the tooth tip deformation.

However, there is no data available for the elastic modulus measured at different frequencies. Moreover, VDI 2736 Part 1 [6], which contains material data and information about material selection, production methods, production tolerances and form design, suggests using the elastic modulus from tensile tests in cases of doubt. However, this does not portray the dynamic loading of a plastic gear during operation.

Further calculation methods for the tooth root stress due to the bending of the plastic teeth are described in the literature. These calculation procedures are adjustments to the Lewis Equation (3). According to the Lewis Equation (3), the strength of a gear tooth can be determined as shown below [7]:

(3) $$W_{s}=s\cdot p\cdot F\cdot y$$

where $W_{s}$ describes the safe bending load of the tooth, s is the safe working stress of the material, p is the circular pitch of the gear, F is the face width of the gears and y is the tooth form factor.

Walton and Shi [8] compared different rating procedures that contain correction factors for lubrication, humidity and temperature, for example. Due to the differences in the correction factors used, the rating procedures show wide discrepancies. Additionally, the lack of experimental data makes the assessment of these rating procedures difficult.

In conclusion, it can be stated that analytic calculations of the tooth deformation and the resulting root stresses can only simplify describing the actual tooth deformation of plastic gears during operation. Actual experimental tooth deformation data for validation is lacking.

A simulative approach using finite element analysis [9] shows that the bending stress in the tooth root area decreases to approximately 2/3 of the theoretical bending stress due to more pronounced load sharing as a result of the tooth deformation. However, the elastic tooth deflection leads to high-stress peaks at unfavorable flank pressure-velocity combinations that are up to seven times higher than the theoretical value at the end of the meshing cycle.

Experiments for the tooth deflection and the rotational delay of the steel gear performed by Terashima, Tsukamoto and Shi [10] show that 98% of the tooth deflection of a steel-plastic gear set is caused by the plastic gear, leading to a rotational delay of the steel gear. This causes severe wear in the dedendum area of the plastic gear’s teeth. The tooth deflection is analyzed by measuring the angular displacement between a steel and plastic gear under load.

However, the test setup used by Terashima, Tsukamoto and Shi [10] does not represent tooth meshing during rotation. Therefore, influences of progressive tooth flank wear, rotational speed and temperature changes on tooth deflection are not taken into account.

As summarized, mostly analytical and simulative approaches exist in the literature. However, there are considerably fewer experimental studies, especially for tooth deformation during operation.

### 1.2. Current Methods and Test Rigs for the Tooth Deformation Measurement during Operation

In general, two main categories of in situ gear test rigs can be distinguished: test rigs for condition monitoring and test rigs for wear and deformation measurement. Condition monitoring aims for the efficient planning of maintenance and the mitigation of downtime due to failure of machine elements [11]. These test rigs use censored data to predict failure in mechanisms and remaining lifetime [12]. Numerous studies focus on the detection of acoustic and vibrational signals as an expression of typical types of gear failure, such as cracks [13,14], tooth flank wear [13,15] and pitting [13,16]. Other sensors, such as oil wear debris sensors [11] and thermographic cameras [17], are less commonly used.

Since condition monitoring test rigs typically do not directly quantify the underlying process of gear failure, such as deformation, there are in situ gear test rigs particularly designed for deformation and wear measurement.

Tooth deformation and wear are measured by using tactile sensors [18], quantifying the weight loss of the plastic gear [19], evaluating the deflection of the plastic gear on a loaded rotatable block [20] or using high-speed cameras and digital image correlation [21]. However, tactile measurements require sufficient accessibility of the measurement point, which might not be the case for the tooth flank of smaller plastic gears. Moreover, the test run has to be stopped for measurement. Gravimetric approaches only enable the quantification of wear if the wear debris leaves the system, and deformations cannot be detected at all. A major limitation of the use of a rotating block is that overlaid effects such as deformation and wear cannot be measured separately. The use of high-speed cameras seems to be the most promising. Nevertheless, the resolution deteriorates with increasing gear speed.

Considering the limitations of current in situ gear test rigs for measuring the tooth deformation during operation, a new testing principle has been developed at the LKT. The principle is described in Section 1.3.

### 1.3. Functional Principles of the LKT In Situ Gear Test Rig

**Figure 2:**
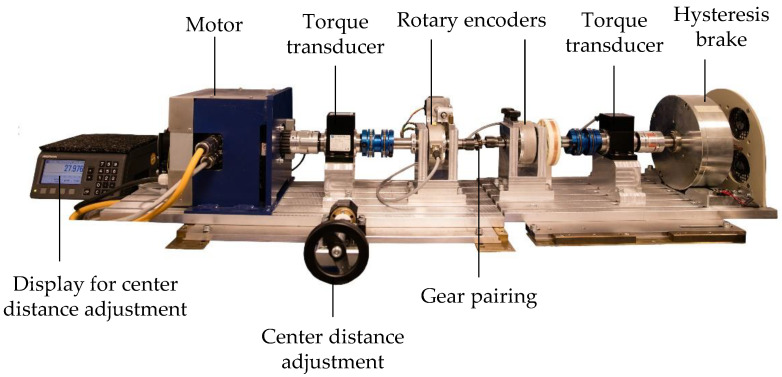
Main components of the in situ gear test rig of the LKT [22].

The main components of the LKT in situ gear test rig (Figure 2) are a three-phase a.c. motor, type DSM150N by Baumüller, Nuremberg, Germany, on the input side, and a hysteresis brake, type CHB-12 by Magtrol, Rossens, Switzerland, on the output side. Torque transducers, type TMB307 by Magtrol, Rossens, Switzerland and rotary encoders, type A020 by Fritz Kübler GmbH, Villingen-Schwenningen, Germany, are located on the input and output shafts.

The motor on the input side drives the shaft on which the steel pinion is mounted. The loading torque is applied to the plastic gear by the hysteresis brake on the output side. Torque fluctuations due to vibrations during the test run and the related frequency spectrum on the input and output sides are measured for the pinion and gear separately by the torque transducers. For continuously tracking the tooth root temperature of the plastic gear, a thermocouple, type K, is placed in a drilled hole with a diameter of 0.6 mm in the tooth root of the plastic gear. Telemetry, type TEL1-PLM-IND, by Kraus Messtechnik GmbH, Otterfing, Germany, is used to transfer the temperature information to a data logging PC. The in situ wear and deformation measurement is based on the rotary encoders on the input and output shafts.

**Figure 3:**
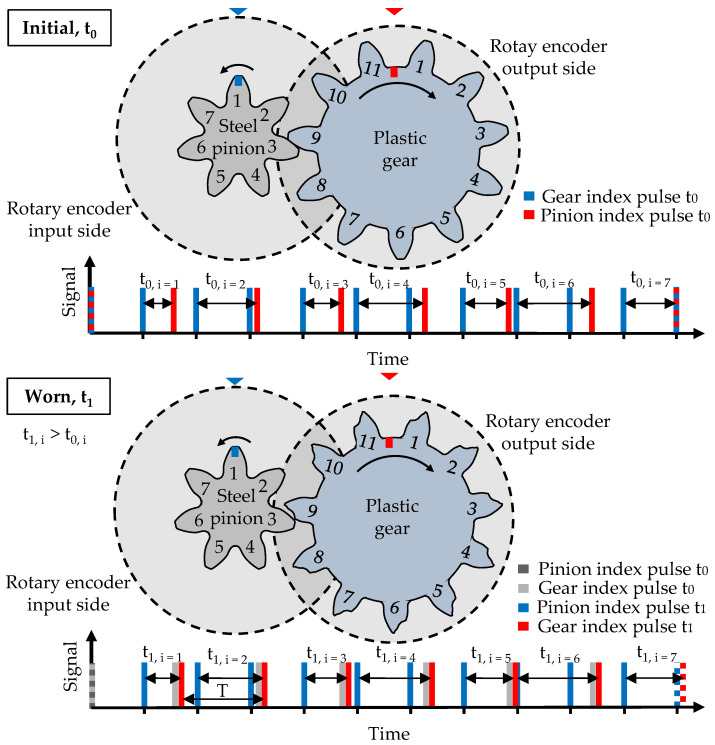
Tooth wear or deformation-induced change in the timing difference between input and output index pulses [23].

The measurement principle developed at the LKT is similar to a single-flank rolling test. However, instead of directly evaluating the angular displacement between pinion and gear, the index pulses of the rotary encoders on the input and output shafts are evaluated. This enables measurement at high operating speeds with high resolution. The idea behind the concept is that progressing wear and deformation of the plastic gear leads to a delay in the index pulse of the plastic gear on the output side. The steel pinion remains unchanged; thus, the time period between the input and output index pulses increases (*t*_0_ < t_1_), as shown in Figure 3.

With the time period T the plastic gear on the output shaft needs to complete one rotation and with the measured time period $\Delta t=t_{1}-t_{0}$ between the two following index pulses of the input and output shafts; the angular displacement Δ*φ* between pinion and gear can be calculated as follows:

(4) $$\Delta \varphi =\frac{∆t}{T}\cdot 360{}^{\circ}$$

The tooth flank wear and deformation Δs can be calculated with the angular displacement Δφ using Equation (5), at a diameter d of choice.

(5) $$\Delta s=\Delta \varphi \frac{\pi \cdot d}{360{}^{\circ}}$$

The total measured tooth deformation consists of elastic deformations and a combination of plastic deformation and wear. Switching between a high loading torque and a low measuring torque enables the separate measurement of the elastic or reversible deformation and the plastic deformation and wear of the plastic tooth [22].

The rotational speed of the plastic gear determines the measurement resolution of the system. At an input speed of 1000 min^−1^, for instance, the plastic gear rotates at approximately 435.9 min^−1^ and consequently needs 0.138 s to complete one rotation. At the given sampling rate of 80 MHz, the encoder is able to sample every 0.0000000125 s, which equals a rotation or angular displacement of 0.00003°. This corresponds to a tooth deformation measurement of ±0.01 µm at the plastic gear’s pitch circle diameter of 39 mm. Therefore, the resolution is high enough to characterize deformations and wear even at high rotational speeds. Other concepts using rotary encoders to directly measure the angle between pinion and gear, on the other hand, might not have a resolution that is high enough for this purpose.

The functional principle of the in situ gear test rig has already been validated in terms of the measurement of tooth flank wear, plastic deformation [24] and elastic deformation [22]. Within this work, the new measurement principle is used to analyze the tooth deformation behavior in dependence on temperature and rotational speed. The results are then compared to the calculated tooth tip deformations according to VDI 2736.

## 2. Materials and Methods

### 2.1. Materials and Specimens

All gear tests were performed using a wire-cut steel pinion created from 100Cr6. The plastic gears were created from PBT Ultradur B4520 by BASF AG, Ludwigshafen, Germany, which is a typical thermoplastic material used for gear applications. In addition, steel gears created from hardened 16MnCr5 were tested according to [22] in order to evaluate the offset of the experimental setup. Since the elastic modulus of steel is considerably higher than the elastic modulus of PBT, it is assumed that any deformation measured using steel instead of plastic gear can be related to measurement offset. The technical data of the investigated pinion and gears according to DIN 867 [25] is shown in Table 1.

The plastic gears were injection molded according to the processing data sheet [26] using an injection molding machine, type 370U-700-30-30, by Arburg GmbH Co. KG, Loßburg, Germany. The main processing parameters are summarized in Table 2. The material has been dried at 100 °C for 4 h before processing.

The plastic gears were clamped between two metal plates in order to measure the tooth deformation. The clamping of the plastic gears used for the in situ gear tests is shown in Table 1.

**Figure 4:**
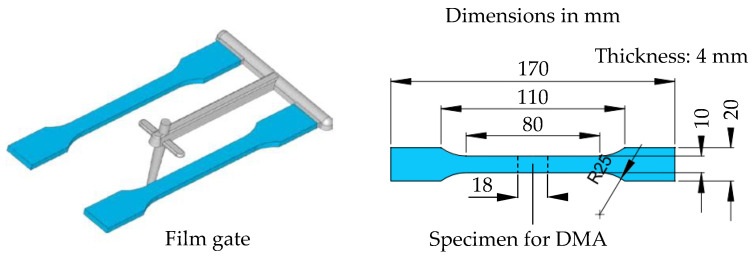
Dimensions and preparation of the DMA specimens.

For the DMA, an injection-molded tensile bar type A according to DIN EN ISO 3167 [27], also created from PBT Ultradur B4520 by BASF AG, Ludwigshafen, Germany, was used. The specimen for the DMA was cut out of the middle of the tensile bar. The dimensions of the tensile bar and the specimen for the DMA are shown in Figure 4, as shown below. The DMA specimen is 18 mm long, 10 mm wide and 4 mm thick.

### 2.2. Testing Methods

#### 2.2.1. In Situ Gear Test Runs

To investigate the tooth deformation behavior in dependence of rotational speed and gear temperature, in situ gear tests were performed. The results were compared to the calculation according to VDI 2736.

To analyze the elastic tooth tip deformation, the high loading torque was used for a time period of 200 load cycles and then reduced to a lower measuring torque of 0.2 Nm, which was applied for 40 load cycles. The difference in deformation was evaluated as the tooth tip deformation λ. One load cycle is defined as 17 rotations of the plastic gear or 39 rotations of the pinion (number of rotations until the same tooth pairing is in contact again). The deformation was measured at the tooth tip diameter d_a_ of the plastic gear, which is 40.373 mm. The high loading torque was set at 0.5 Nm, 0.75 Nm, 1.00 Nm, 1.25 Nm, 1.5 Nm, 1.75 Nm and 2.00 Nm. The rotational gear speed was varied between 1000 min^−1^ and 510 min^−1^ and the gear temperature between 23 °C and 80 °C. For each parameter combination, *n* = 3, gear tests were performed. To calibrate the test setup, gear tests at the mentioned loads and speeds using a steel gear, which is assumed not to be deformed under the tested loads, were conducted in advance. In this case, the measured deformations are measurement offsets. The average value is subtracted from the measured deformations at the plastic gear tests. For the calibration tests, *n* = 3 tests were also performed.

The results were compared to the calculation of the tooth tip deformation λ according to Equation (1). The used torque levels of the gear tests were converted to the corresponding nominal tangential forces of 25.6 N, 38.5 N, 51.3 N, 64.1 N, 76.9 N, 89.7 N and 102.6 N using the pitch circle diameter d_2_ = 39 mm. Since the load is not reduced completely during the in situ gear tests, the calculated deformation at 10.3 N (equals 0.2 Nm) was subtracted from the calculated tooth tip deformations at the different load levels. The temperature was set at 23 ± 2 °C and 80 ± 4 °C using compressed or heated air. For the calculations, the elastic modulus of the steel pinion E_1_ was assumed to be 210.000 N/mm^2^. The elastic moduli E_2_ of the PBT gear at 23 °C and 80 °C were graphically evaluated using Figure 1 according to VDI 2736 Part 1. Hence, E_2_ = 2700 N/mm^2^ at 23 °C and E_2_ = 650 N/mm^2^ at 80 °C were assumed for the elastic moduli.

#### 2.2.2. Dynamomechanical Analysis (DMA)

The DMA was performed under a very small torsional load. The measured deformation was 0.02%. A temperature sweep between 25 °C and 80 °C (in steps of 5 °C) and a frequency sweep between 0 Hz and 20 Hz (in steps of 1 Hz) were conducted to evaluate the thermal-mechanical behavior of PBT Ultradur B4520 in the relevant ranges.

## 3. Results and Discussion

### 3.1. In Situ Gear Test Runs

**Figure 5:**
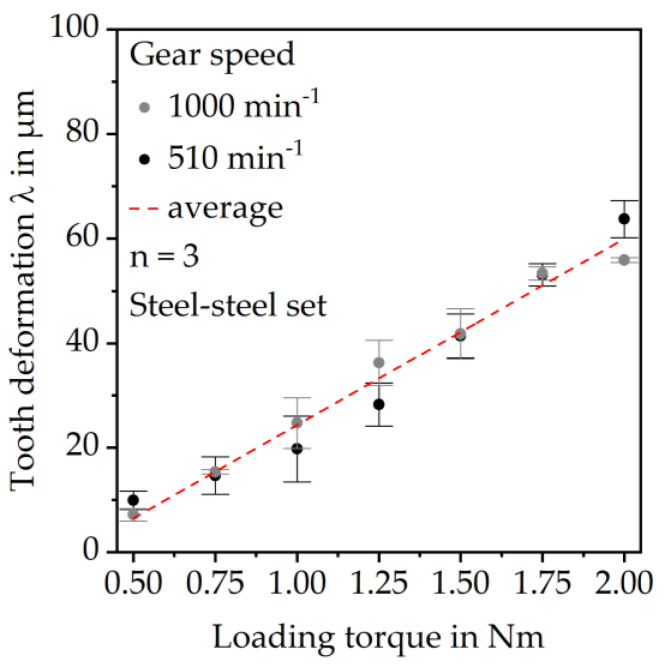
Measurement offset of the in situ gear test rig evaluated at the tooth tip diameter.

The calibration tests with the steel gear show increasing deformation with increasing load. Since the deformation is quite similar for each rotational speed, the average deformation was used for the correction of the results of the plastic gear tests. The measurement offset is a result of the deformation of the test rig components (e.g., shafts and fixations). Figure 5 shows the results of the calibration tests.

**Figure 6:**
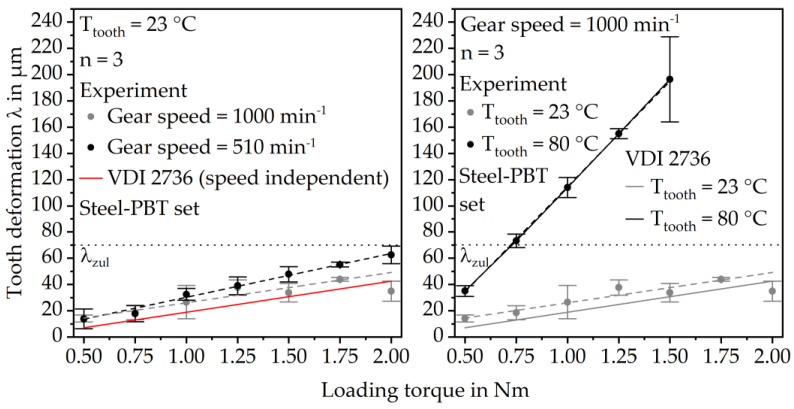
Tooth deformation in dependence of rotational speed (**left**) and gear temperature (**right**) in comparison to the calculated tooth deformation according to VDI 2736.

The results of the in situ gear tests under variations in rotational speed and temperature are summarized in Figure 6. For all speeds and temperatures, the tooth tip deformation increases roughly linearly with increasing load. The effect of temperature on tooth tip deformation is more pronounced than the effect of gear speed. At 23 °C, below the glass transition temperature T_g_ of PBT, and a rotational gear speed of 1000 min^−1^ the tooth tip deformation increases from 14.1 ± 2.6 µm at 0.50 Nm loading torque to 18.5 ± 5.3 µm at 0.75 Nm. At 1.0 Nm loading torque, the tooth deformation is 26.6 ± 12.6 µm. It increases further to 37.7 ± 5.8 µm at 1.25 Nm. After a slightly lower average deformation of 33.8 ± 5.8 µm at 1.5 Nm the deformation increases from 43.9 ± 1.4 µm at 1.75 Nm loading torque to 34.9 ± 7.7 µm at 2.00 Nm. Considering the standard deviations, the tooth deformation increases roughly linearly with increasing loading torque. At a tooth temperature of 80 °C above T_g_, the tooth tip deformation at 0.50 Nm loading torque is 35.0 ± 4.1 µm. It increases up to 196.4 ± 32.4 µm at 1.50 Nm loading torque. At even higher torques of 1.75 Nm and 2.00 Nm the gears fail before the end of the test run due to tooth root breakage. However, microscopy shows no signs of initial cracks in the tooth root area at 32 × magnification after the tests at 80 °C and 1.50 Nm loading torque. The comparison with the calculation of the tooth tip deformation according to VDI 2736 (Equation (1)) shows very good consistency with the experimental data at 80 °C. At 23 °C, the calculated deformation is also quite similar to the experimentally measured deformation. The load-dependent increase rate is roughly identical. The calculated deformation is slightly lower, but mostly within the range of the standard deviations of the experiments.

At a rotational gear speed of 510 min^−1^ the average tooth tip deformation is 13.8 ± 7.5 µm, which is approximately the same as at 1000 min^−1^ rotational speed. However, it increases roughly linearly up to 62.9 ± 6.7 µm, which is approximately 50% higher than the calculated results and significantly higher than the deformation at 1000 min^−1^ gear speed. Moreover, the deformation is already within the range of the maximum allowed deformation λ_zul_ = 70 µm according to VDI 2736 part 2 (Equation (2)) at a loading torque of 2.00 Nm.

### 3.2. Dynamomechanical Analysis (DMA)

**Figure 7:**
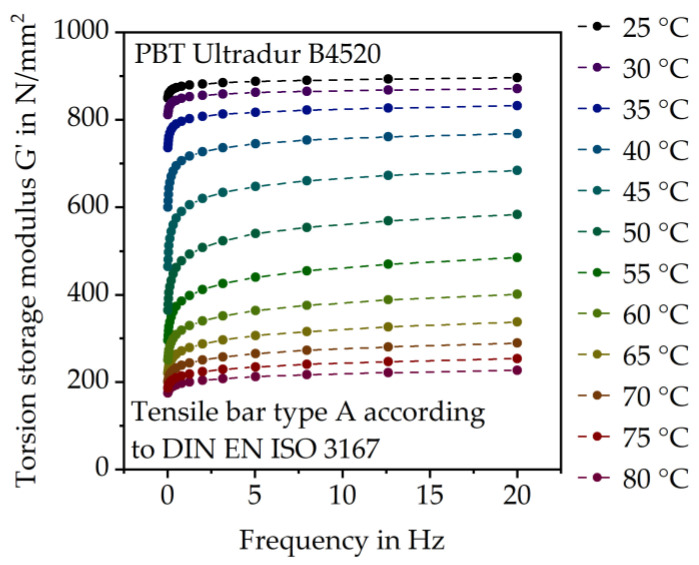
Torsion storage modulus G’ of PBT Ultradur B4520 depending on the load frequency at different temperatures.

The outcome of the gear tests corresponds well to the results of the DMA (Figure 7). The influence of temperature on the elastic modulus is higher than the influence of loading speed, which is consistent with the literature [2].

**Figure 8:**
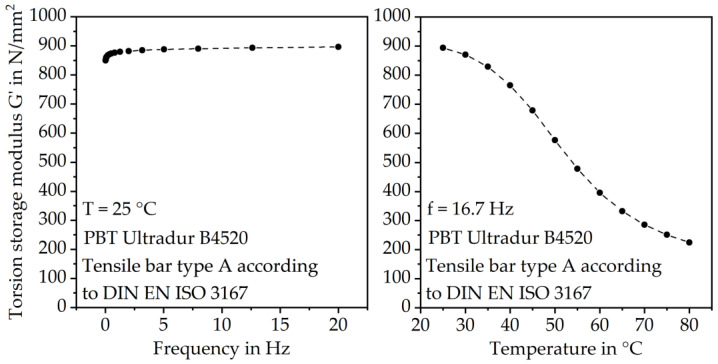
Torsion storage modulus G’ in dependence of frequency at 25 °C (**left**) and temperature at 16.7 Hz load frequency (**right**).

With increasing temperature, the torsion storage modulus G’, which corresponds to the elastic modulus, decreases severely, according to the DMA results. G’ increases with higher frequency. The effect of the load frequency on G’ is especially prominent at low frequencies between 0 Hz and 3 Hz and around the glass transition temperature T_g_ (between 40 °C and 60 °C). The DMA results also show that the effect of temperature on the elastic material behavior is significantly higher than the effect of frequency, as shown in Figure 8.

At 8.5 Hz (510 min^−1^) G’ is at approximately 890 N/mm^2^ and increases only slightly to approximately 894 N/mm^2^ at 16.7 Hz (1000 min^−1^). The temperature increase from 23 °C to 80 °C causes a severe reduction in G’ from 894 N/mm^2^ to approximately 224 N/mm^2^.

However, even the small increase in G’ at the investigated frequencies leads to significant changes in tooth deformation, as shown by the results of the gear tests.

## 4. Conclusions

The tooth deformation of plastic gears acts as a pitch error and therefore influences the running noise and lifetime of plastic gears. Consequently, it should be taken into account when designing plastic gear sets. However, no experimental results for the frequency- and temperature-dependent tooth deformation behavior of plastic gears exist in the literature until now.

Hence, within this work, a new test rig design, which enables a more comprehensive understanding of the tooth deformation of plastic gears during operation, was used to investigate the influence of rotational speed and temperature on tooth deformation. Moreover, the results of the in situ gear tests were compared to the calculated deformations according to VDI 2736 part 2.

The results of the gear tests show that the effect of temperature on tooth deformation is much higher than the effect of rotational speed. However, at lower rotational speeds, the tooth tip deformation is much higher (62.9 ± 6.7 µm at 2.00 Nm loading torque) than at higher speeds (34.9 ± 7.7 µm at 2.00 Nm loading torque). In addition, the actual tooth deformation is significantly higher (roughly 50%) than the calculated one at lower speeds and high loads. In this case, the actual tooth deformation is already within the range of the maximum allowed deformation at high loads.

Current and future research at the LKT focuses on the effect of the injection molding process on the crystallinity of the plastic gear’s teeth and the interaction with temperature- and frequency-dependent tooth deformation behavior. This research aims for an improved calculation formula for tooth tip deformation.

## Figures and Tables

**Table 1:** Technical specifications of the investigated gear sets.

Pairing According to DIN 867	Steel Pinion	Plastic Gear	Steel Gear
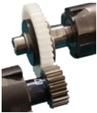	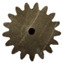	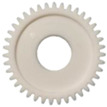	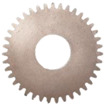
Material	Hardened 100Cr6	PBT Ultradur B4520	Hardened 16MnCr5
Module	1 mm		
Pressure angle	20°		
Number of teeth	17	39	
Gear width	8 mm	6 mm	
Profile shift	0.2045 mm	−0.3135 mm	
Pitch circle diameter d_2_	17 mm	39 mm	
Tip diameter d_a_	19.409 mm	40.373 mm	

**Table 2:** Main processing parameters for the injection molding of the plastic gears.

Processing Parameter	Parameter Setting
Screw diameter	18 mm
Mass temperature	260 °C
Mold temperature	60 °C
Injection/Holding/Cooling/Cycle time	2.2 s/6 s/25 s/42.8 s
Holding pressure	600 bar
Cylinder temperature profile (Nozzle → indentation)	260 °C/250 °C/240 °C/230 °C/90 °C

## Data Availability

Not applicable.

## References

[1] Singh A.K., Singh S., Singh P.K. (2017). Polymer spur gears behaviors under different loading conditions: A review. J. Eng. Tribol..

[2] Ehrenstein G.W. (2007). Mit Kunststoffen Konstruieren: Eine Einführung.

[3] Feulner R. (2008). Verschleiß Trocken Laufender Kunststoffgetriebe: Kennwertermittlung und Auslegung. Ph.D. Thesis.

[4] (2016). Thermoplastic Gear Wheels—Cylindrical Gears: Calculation of the Load-Carrying Capacity.

[5] Illenberger C. (2022). Zahnflankentragfähigkeit Ölgeschmierter Kunststoffverzahnungen. Ph.D. Thesis.

[6] (2016). Thermoplastic Gear Wheels: Materials, Material Selection, Production Methods, Production Tolerances, Form Design.

[7] Lewis W. (1893). Investigation of the Strength of Gear Teeth. Proceedings of the Engineers Club of Philadelphia.

[8] Walton D., Shi Y.W. (1989). A comparison of ratings for plastic gears. Proc. Inst. Mech. Eng. Part. C Mech. Eng. Sci..

[9] Van Melick H.G.H. (2007). Tooth-bending effects in plastic spur gears. Gear Technol..

[10] Terashima K., Tsukamoto N., Nishida N. (1986). Development of plastic gears for power transmission: Design on load-carrying capacity. Bull. JSME.

[11] Shah H., Hirani H. (2014). Online condition monitoring of spur gears. Int. J. Cond. Monit..

[12] Martin G., Vogel S., Schirra T., Vorwerk-Handing G., Kirchner E. Methodical Evaluation of Sensor Positions for Condition Monitoring of Gears 2018. Proceedings of the DS 91: NordDesign 2018.

[13] Yao Y., Wang H., Li S., Liu Z., Gui G., Dan Y., Hu J. (2018). End-to-end convolutional neural network model for gear fault diagnosis based on sound signals. Appl. Sci..

[14] Loutas T.H., Sotiriades G., Klaizoglou I., Kostopoulos V. (2009). Condition monitoring of a single-stage gearbox with artificially induced gear cracks utilizing on-line vibration and acoustic emission measurements. Appl. Acoust..

[15] Figlus T., Stanczyk M. (2014). Diagnosis of the wear of gears in the gearbox using the wavelet packet transform. Metalurgija.

[16] Hu C., Smith W.A., Randall R.B., Peng Z. (2016). Development of a gear vibration indicator and its application in gear wear monitoring. Mech. Syst. Signal Process..

[17] Resendiz-Ochoa E., Saucedo-Dorantes J., Benitez-Rangel J., Osornio-Rios R., Morales-Hernandez L. (2019). Novel Methodology for Condition Monitoring of Gear Wear Using Supervised Learning and Infrared Thermography. Appl. Sci..

[18] Sosa M., Björklund S., Sellgren U., Olofsson U. (2015). In situ surface characterization of running-in of involute gears. Wear.

[19] Yousef S.S., Burns D.J., McKinlay W. (1973). Techniques for assessing the running temperature and fatigue strength of thermoplastic gears. Mech. Mach. Theory.

[20] Hooke C.J., Mao K., Breeds A.R., Kukureka S.N. (1993). Measurement and Prediction of the Surface Temperature in Polymer Gears and Its Relationship to Gear Wear. J. Tribol..

[21] Černe B., Petkovšek M. (2022). High-speed camera-based optical measurement methods for in-mesh tooth deflection analysis of thermoplastic spur gears. Mater. Des..

[22] Herzog C., Drummer D. (2023). Test Rig for the In Situ Measurement of the Elastic Tooth Deflection of Plastic Gears. Polymers.

[23] Herzog C., Wolf M., Drummer D. (2022). In Situ Measured Tooth Flank Wear of Plastic Gears under Spectrum Loading. Polymers.

[24] Herzog C., Wolf M., Schubert D., Drummer D. (2022). In situ investigation of the influence of varying load conditions on tooth deformation and wear of polymer gears. Forsch. Ingenieurwes.

[25] (1986). Bezugsprofile für Evolventenverzahnungen an Stirnrädern (Zylinderrädern) für den Allgemeinen Maschinenbau und den Schwermaschinenbau.

[26] BASF SE (2022). CAMPUS Data Sheet PBT Ultradur® B 4520, Frankfurt. https://www.campusplastics.com/material/pdf/180906/UltradurB4520?sLg=de.

[27] (2014). Kunststoffe—Vielzweckprobekörper.

